# I know what i like when i see it: Likability is distinct from pleasantness since early stages of multimodal emotion evaluation

**DOI:** 10.1371/journal.pone.0274556

**Published:** 2022-09-13

**Authors:** Marianne Tiihonen, Thomas Jacobsen, Niels Trusbak Haumann, Suvi Saarikallio, Elvira Brattico

**Affiliations:** 1 Center for Music in the Brain, Department of Clinical Medicine, Aarhus University & The Royal Academy of Music Aarhus/Aalborg, Aarhus, Denmark; 2 Experimental Psychology Unit, Humanities and Social Sciences, Helmut Schmidt University / University of the Federal Armed Forces Hamburg, Hamburg, Germany; 3 Department of Music, Art and Culture Studies, University of Jyväskylä, Jyväskylä, Finland; 4 Department of Educational Sciences, Psychology, Communication, University of Bari Aldo Moro, Bari, Italy; Public Library of Science, UNITED KINGDOM

## Abstract

Liking and pleasantness are common concepts in psychological emotion theories and in everyday language related to emotions. Despite obvious similarities between the terms, several empirical and theoretical notions support the idea that pleasantness and liking are cognitively different phenomena, becoming most evident in the context of emotion regulation and art enjoyment. In this study it was investigated whether liking and pleasantness indicate behaviourally measurable differences, not only in the long timespan of emotion regulation, but already within the initial affective responses to visual and auditory stimuli. A cross-modal affective priming protocol was used to assess whether there is a behavioural difference in the response time when providing an affective rating to a liking or pleasantness task. It was hypothesized that the pleasantness task would be faster as it is known to rely on rapid feature detection. Furthermore, an affective priming effect was expected to take place across the sensory modalities and the presentative and non-presentative stimuli. A linear mixed effect analysis indicated a significant priming effect as well as an interaction effect between the auditory and visual sensory modalities and the affective rating tasks of liking and pleasantness: While liking was rated fastest across modalities, it was significantly faster in vision compared to audition. No significant modality dependent differences between the pleasantness ratings were detected. The results demonstrate that liking and pleasantness rating scales refer to separate processes already within the short time scale of one to two seconds. Furthermore, the affective priming effect indicates that an affective information transfer takes place across modalities and the types of stimuli applied. Unlike hypothesized, liking rating took place faster across the modalities. This is interpreted to support emotion theoretical notions where liking and disliking are crucial properties of emotion perception and homeostatic self-referential information, possibly overriding pleasantness-related feature analysis. Conclusively, the findings provide empirical evidence for a conceptual delineation of common affective processes.

## Introduction

Emotions are complex phenomena with subjective experiential and conceptual components, often accompanied by neurophysiological changes in the whole organism [1]. The dimensions of liking, pleasantness, valence and preference underlie many emotion theories or are frequently used in empirical settings as well as in everyday language when dealing with emotions [2–6]. Despite likability and pleasantness being seemingly similar terms, applying them interchangeably could lead to conceptual and terminological inaccuracy. A good example demonstrating the distinction between liking and pleasantness is a situation where a person enjoys or likes something that, based on its sensorial, representational or semantic features, is unpleasant or otherwise low in valence, but nevertheless is being enjoyed: In music, acoustic properties corresponding to happy or sad features have been shown to underlie correspondingly different neural correlates [7]. Also, regarding music induced enjoyment in particular, three preconditions have been proposed to be crucial for sadness to be enjoyed: Sadness should not be perceived as threatening, it should be aesthetically pleasing, and it should contribute to psychological wellbeing, for example, in the form of mood regulation [8]. In cinematography, a complex emotional state of being moved has been shown to mediate negative emotions and the resulting enjoyment [9]. Furthermore, in the literature of empirical aesthetics, a recent model has suggested that the enjoyment of negative emotions in art is enabled by shifts between the psychological mechanisms of distancing and embracing which are activated by cognitive schemata of art, representation or fiction [10]. According to this model, this activation shift between distancing and embracing enables the delicate balance of safely enjoying something that in another context would not be enjoyable, rather than enjoying the negative emotion, such as sadness, itself [11–13]. Furthermore, a recent study has shown that the enjoyment of sadness, in particular, takes place when it first leads to the state of being moved, again referring to the idea that the negative emotions may employ a mediating mechanism to be enjoyed [14]. This apparent contradiction, liking or preferring something that is unpleasant or low in valence, seems fairly common, being part of cognitive functions, such as attention and memory [10], cognitive schemata [12] as well as affect-related mechanisms, such as rumination, emotion regulation and well-being in general [15–18].

Next to the above presented psychological models and mechanisms, there is empirical evidence for separating liking from pleasantness. A functional magnetic resonance imaging study demonstrated an anatomy-functional separation for liking (particularly in deep limbic structures) and emotion perception (mainly in frontotemporal regions), supporting the ability to like something unpleasant, such as sadness [7]. Furthermore, a music rating study showed that preference rating was not a particularly successful predictor of different labels describing emotions, whereas pleasantness, in combination with arousal and familiarity, was more accurate at predicting the corresponding emotions [5].

Regarding representational dimension of affect, it has been shown that semantically non-representational visual and acoustic stimuli can be used to convey affective information in a short time of less than one second. Nevertheless, based on a model on aesthetic chronometry [19] an initial feature analysis has been postulated to be the foundation of an aesthetic process, which then is immediately followed by an early affective reaction. Furthermore, affective priming protocols have been used to test whether musical chords with different valence qualities, as usually associated with varying degrees of consonance and dissonance, would activate unintentionally either positive or negative affective processing, thus determining the reaction times to the affective judgement of the acoustic target stimuli [20, 21]. The results of both studies showed that single chords as well as chord sequences of different valence qualities primed the reactions to the targets, such that the targets were evaluated faster and more correctly in congruent prime-target pairs. Similarly, simple visual elements, such as geometric patterns, convey affective information. Studies applying geometric patterns with varying degrees of complexity and symmetry can be used to predict which kinds of patterns are considered most pleasing. Despite differences in the individual judgement styles, across all participants, symmetry of novel, non-representational graphic patterns have been found to be the best predictor of high beauty ratings among non-experts, secondary predictor being the complexity of the patterns [22, 23]. For terminological clarity, henceforth we use the term *representational* to refer to affective content which conveys semantic meaning (such as the here applied primes depicting real-life sceneries), and the term *non-representational* is used to refer to abstract affective information conveyed by aesthetic stimuli (such as the here applied target patterns and musical chords) [24].

The main motivation of this study was to find out whether a temporal separation of the seemingly overlapping affective processes underlying liking and pleasantness takes place already during the rather early perceptual process. These early, bottom-up processes of initial affective reactions were targeted by applying the above mentioned affective priming protocol. Although affective priming has primarily been studied within a single modality, it has been shown to be a robust and reproducible phenomenon across sensory modalities, tested with various priming stimuli, tasks as well as target stimuli [25]. Affective priming effect can be observed behaviourally by inspecting the reaction times of the participants while they answer target-related questions and by varying the congruency of the prime and the target pairs. Should an affective priming effect take place, the response time to the congruent prime-target pairs can be expected to be shorter than to the incongruent pairs [25]. Here, affective priming is applied cross-modally and bi-directionally. Cross-modality means that the prime and target stimuli always are of two different modalities, resulting in cross-modal pairs in varying order, visual prime stimuli being followed by acoustic target stimuli, and the other way round. Thus, bi-directionality means that the priming effect can take place from vision to audition or from audition to vision. Applying affective priming in a cross-modal setting allows the investigation of whether psychological mechanisms of liking and pleasantness ratings are modality specific between the two sensory modalities, or whether they are shared across the modalities. Based on the literature above, we argue that the affective separation of pleasantness from liking occurs already in the early stages of stimulus perception, and that it depends on the stimulus features. As demonstrated above, it seems that liking and pleasantness ratings in both domains are separate processes, thus a difference between the tasks across the modalities is expected. Furthermore, it is hypothesized that the feature detection-based pleasantness rating would be faster than the liking rating. Due to the combination of semantically representational (prime) and non- representational (target) cross-modal stimuli, the difference between the modalities remains exploratory. Nevertheless, a bi-directional cross-modal affective priming effect is expected to take place.

## Method

### Participants

The participants were healthy, right-handed adults, mean age 26.8 (SD = 5.0), ten women and eight men (one did not specify the gender), and they had normal hearing and normal or corrected-to-normal vision. None of the individuals suffered from neurological or psychiatric disorders, they were not under medication, nor did they have severe physical limitations. Furthermore, professional musicians and art experts, defined as conservatory or art history students, were excluded. The data from one participant was excluded due to a technical failure, so finally 19 participants were included in the statistical analysis. Eleven of the participants were university students, three were full-time employed, one was unemployed, and four did not specify their current occupation. The participants were recruited via an announcement for the study posted on a social media platform. The study was conducted according to the Helsinki Declaration II, and it was part of a study granted ethical approval of the Ethics Committee of Central Region Denmark (55441). All participants were provided with written information on the experiment, and they also had the possibility to contact the experimenters by e-mail or by telephone for questions concerning the experimental procedure. Participation in the study was voluntary, and as a compensation for their time, the participants received a voucher with a value of 100 Danish Kroner for online shopping. A signed consent form was required from everyone before the start of the experiment.

### Stimuli

The present study used visual and acoustic prime and target stimuli. All the stimuli had been demonstrated to evoke either distinctively positive or negative affect in previous experiments without distinguishing between liking and pleasantness. The acoustic target stimuli were musical chords demonstrated to convey positive affect (high on valence, happiness and liking, mainly consonant major chords) and negative affect (low on valence, happiness and liking, mainly dissonant or minor chords) [26, 27]. The visual target stimuli were black and white patterns, validated according to their aesthetic value (beautiful, not beautiful), and symmetry and complexity [22], thus representing positive (beautiful, optimal symmetry and complexity) or negative valence (not beautiful, asymmetric and complex). The visual prime stimuli were from the databank of the International Affective Picture System (IAPS) and had been validated to have medium arousal (*mean* 5.4) and high valence (*mean* 7.2) (e.g., rewarding moments), or low valence (*mean* 2.5) (e.g., accidents, illness, violence) [28]. Similarly, the acoustic primes were from the databank of the International Affective Digital Sounds (IADS), and the same criteria of medium arousal (*mean* positive 5.9, *mean* negative 6.7) and high (*mean* 6.8) and low (*mean* 2.6) valence were applied for the acoustic stimuli as well. Unlike the target stimuli, both primes were semantically representational, thus they had either a culturally learned or biologically relevant positive or negative representation (e.g., sounds and images of cute animals or a visual close-up of an infected eye) [29]. In total there was 160 different stimuli, consisting of 20 positive and 20 negative acoustic targets, 20 positive and 20 negative visual targets, 20 positive and 20 negative acoustic primers, and 20 positive and 20 negative visual primers. The target stimuli were chosen such that their valence and arousal ratings would be as similar as possible across the modalities. This was based the ratings provided by the authors who created the IAPS and IADS stimuli [28, 29]. The mean valence and arousal ratings of the target stimuli, including original data bank identifiers, as well as further details on the prime stimuli can be found in the S1 to S5 Tables, S1 and S2 Figs. As the IADS sounds are 6000 ms long, they were shortened for the purpose of the study by either cutting from the beginning or from the end of the sample depending on which editing retained the recognizability of the semantic information of the IADS sounds. The editing was based on the evaluation of two of the authors, and it was conducted with the sound editing program Audacity [30].

### Procedure

#### Trial structure

A cross-modal affective priming protocol was applied to investigate whether affective processing depends on the sensory modality (auditory or visual) or the given affective rating task (liking or pleasantness). Also, it was investigated whether congruent valence between primes and targets (i.e., positive prime and target, or negative prime and target) resulted in faster responses than incongruent valence (i.e., positive prime and negative target, or negative prime and positive target). Indicating the start of the experimental trial, a visual fixation cross was presented for 500 ms, followed by a prime (IAPS picture or IADS sound) presented for 1500 ms. The target followed the prime with a stimulus onset asynchrony (SOA, the time between the onset of the prime and the onset of the target) of 300 ms, and it was presented for 1200 ms. This means that the prime was presented alone for 300 ms and was then followed by the target for additional 1200 ms, resulting in an overall prime duration of 1500 ms. A similar trial structure, where primes and targets partially overlap, has been successfully applied in another affective priming studies applying musical chords [21]. The SOA has been shown to be effective ranging from 0 to 300 ms in affective priming studies, such that the shorter the SOA, the more effective the priming effect [25, 31]. Prime and target pairs always resulted in a cross-modal combination. The longer SOA of 300 ms and the simultaneous presentation of the prime and target was to compensate for the temporal differences in stimulus presentation across the two sensory modalities, and secondly, to ensure that participants had sufficient time to perceive the prime, as the IADS and IAPS primes represented natural sceneries, e.g., an image of an accident or a sound of a slap followed by a cry. The target duration of 1200 ms was deemed sufficient as the stimuli are perceptually rather simple, and furthermore 1200 ms had been shown to be sufficient in a previous similar design where an evaluative judgement was requested for the here used visual patterns [32].

**Fig 1:**
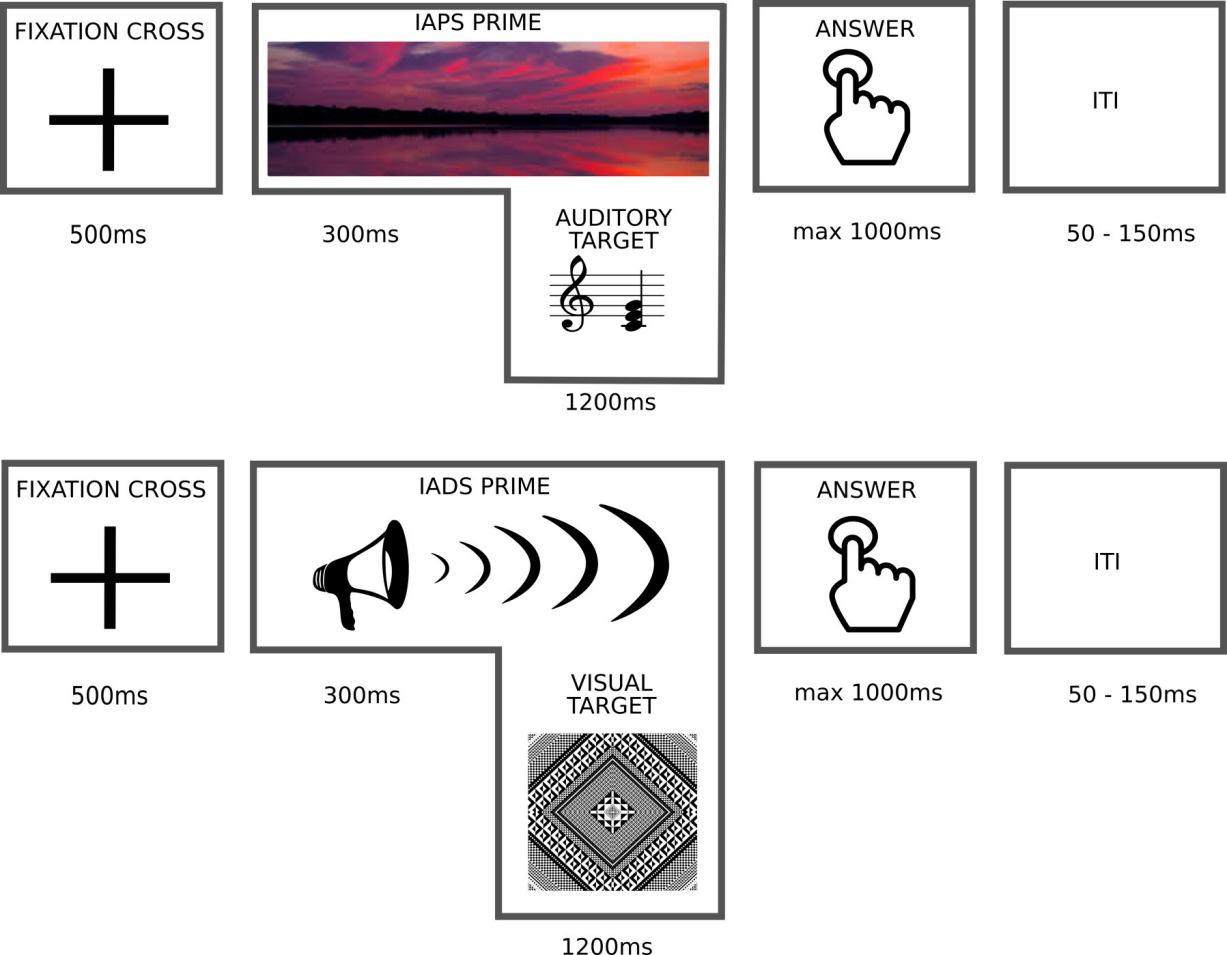
Trial structure for both types of prime-target pairs. Upper diagram: A single trial of the condition 1 with a visual prime and acoustic target. Lower diagram: A single trial of the condition 2 with an acoustic prime and visual target.

*Affective rating task*. The task was to rate whether participants liked the target or not or whether they thought the target was pleasant or unpleasant. All prime-target combinations were rated on both liking and pleasantness (i.e., presented twice with the alternating task). Participants were informed about the difference between liking and pleasantness judgement, such that the term pleasantness was told to refer to the assessment of the perceptual features of the stimuli, whereas liking was told to refer to one’s personal judgement with no right or wrong answers. Also, it was explained that it was possible that liking and pleasantness evaluations contradict, such that one does not like a pleasant stimulus, and the other way round. Providing the answer to the rating task was possible immediately upon the appearance of the target stimulus. Nevertheless, a separate answering cue was prompted 1200 ms after the target stimulus during which the participants were asked to give their affective judgement of the target. A separate text prompt on the screen was provided at the beginning of each block to inform the participants whether a liking or a pleasantness judgement was requested. In addition, immediately prior to providing the answer, participants were explicitly asked for a liking or pleasantness judgement. The answer was provided via a button press indicating whether the participants either liked the target or not, or whether they thought the target was either pleasant or unpleasant in a two-alternative forced choice. These responses were recorded in milliseconds and used for the statistical analysis. The answering cue was presented for 1000 ms, meaning that the participants had altogether 2200 ms answering time. If the participants still provided their answer after the end of the trial, the answer was recoded nevertheless, but these answers were excluded in the analysis. An inter trial interval (ITI) varying between 50 ms to 150 ms, with equal distribution around mean 100 ms, followed between the trials. The ITI was randomized computationally (Fig 1).

### Experimental design

The experiment consisted of two short practice blocks and four main experimental blocks (see S6 Table for the session structure). Each block contained 40 trials that consisted of the 20 positive and 20 negative prime and target stimuli. Between the prime and the target stimuli, 20 trials were assigned with congruent valence and 20 trials with incongruent valence. The combination and order of the specific prime and target stimulus was randomized for each participant. Each prime-target combination was presented twice, once for each of the two affective judgment tasks. The change of the block was announced with new instructions referring either to the change of the stimulus modality or the task. Thus, all in all the experiment consisted of 160 trials. The blocks were presented in random order for each participant.

#### Session procedure

The experiment took place in the facilities of Center for Music in the Brain (MIB) at Aarhus University. Participants were seated in a quiet and dimly lit room in front of a table with a keyboard, computer screen and a mouse. The Presentation software (Neurobehavioral Systems, Ltd.) was used for presenting the stimuli and recording the responses. The visual stimuli were centred on a 16” laptop screen and presented in a fixed height of 400 pixels in a screen resolution of height 600 pixels and width 800 pixels with 32-bit colour resolution. The acoustic stimuli were presented to the participants via headphones (AKG K271) in stereo at a sampling rate of 44.1 kHz 16-bit resolution. The volume was the same for each participant; nevertheless, prior to the beginning it was confirmed by asking the participant whether the sound level was appropriate. After receiving oral instructions, the participants practiced giving their ratings in order to acquaint themselves with the tempo of the protocol, with shifting their focus between acoustic and visual targets and practicing the distinction of liking and pleasantness ratings. All in all, the participants gave twenty practice ratings, five for each modality and each task combination. Before the start of the experiment, the participants were provided with the opportunity to ask questions. Between each block the participants had the opportunity to have a break of a few minutes before continuing with the experiment. Altogether the experiment lasted about 60 minutes.

### Statistical analysis

The aim of the statistical analysis was to find out whether there was a difference in participants‘ response times (RT) to the given affective judgement tasks. In particular, it was tested whether the RT depended on the sensory modality (auditory / visual), on the given task (liking / pleasantness) and on the congruency (congruent / incongruent), and their possible interaction. Linear mixed modelling (LMM) with maximum likelihood ratio testing and estimated marginal means (emmeans) was deemed as the most appropriate analysis for the response latencies. Linear mixed modelling was chosen over fixed effect as it allows the treatment of possible variability within the subjects as a random effect by assuming that each participant can have a different baseline [33].The analysis was conducted using R (Version 1.1.423) [34] and R Studio software environment [35] for statistical computing, using the *lmer* function from the *lme4* Package (Version 1.1–21). The LMM with the maximum likelihood test are based on the idea of fitting a model on the data by first creating the simplest model that theoretically could explain the obtained results. Subsequently, the model complexity is increased by adding possible categorical co-variates to the model, while further testing the fit of the model using the LMM. Increasing the model complexity with the test parameters of congruency, task and modality took place as follows: First, it was tested whether the obtained data could be explained by the random variability of the subjects by using it as the sole factor (model 1). Then the factor congruency was added (model 2), and further the factor task (model 3) and modality (model 4) were fitted on the data, always treating the subject as a random effect and the other variables as fixed effects. Finally, interaction terms were added between the fixed effects to test whether an interaction existed between congruency and task (Model 3i), task and modality (Model 4i), or between congruency, task, and modality (Model 4ib) in the most complex model. Subsequently, the model comparison was done by applying an ANOVA with a Chi-square likelihood ratio test from the *lmerTest* package (Version 3.1–0) [36] to test which of the created models would demonstrate the highest probability in explaining the data. To estimate the significance of the models, p-values for each factor of the models were computed using the Satterthwaite method as this has been reported to be best suitable for mixed effect models of varying sample sizes [37]. Finally, the estimated marginal means and the corresponding contrasts for the model with the best prediction accuracy were computed using the *emmeans* package from R (Version 1.4) [38]. Marginal means represent the mean response times for each category of a factor, allowing a direct comparison of the tested variables of the model.

## Results

In the statistical analysis, single outlier data points, defined as response latencies of 50 ms or less and response latencies equal to 2200 ms or more, were removed. The lower threshold of 50 ms was interpreted as a mistake, as there were only few answers given in less time than that, and the rest of the answers indicated clearly longer response latencies. The upper boundary was set to 2200 ms, as no single trial lasted longer than that. The outlier removal resulted in removing one data point at the lower threshold of 50 ms and 42 datapoints at the upper threshold of 2200 ms. The summary statistics of the response times after the removal of the outliers are enlisted in Table 1.

**Table 1:** Summary statistics of the response times.

Response times				
Task	Modality	N	M	SD
Liking	Auditory	744	959	423
Pleasantness	Auditory	750	994	423
Liking	Visual	751	912	372
Pleasantness	Visual	733	994	424

Response times in milliseconds to each task within both modalities.

N = number of observations, M = means response time, SD = Standard deviation of the response time.

After the outlier removal, the following linear mixed models were fitted on the data: Model 1: RT ~ (1 | ID), Model 2: RT ~ Congruency + (1 | ID), Model 3: RT ~ Congruency + Task + (1 | ID), Model 3i: RT ~ Congruency * Task + (1 | ID), Model 4: RT ~ Congruency + Task + Modality + (1 | ID), Model 4i: RT ~ Congruency + Task * Modality + (1 | ID), and Model 4ib: RT ~ Congruency * Task * Modality + (1 | ID). Each model, compared to the previous model, is increased in its complexity either in the form of an added co-variate or an interaction term (see the S7 to S13 Tables for the detailed results of each model).

Of all the above enlisted models, models 1, 2, 3, and 4i showed significant effects for their corresponding co-variates. The subsequent Chi square likelihood ratio tests with t-statistics indicated that Model 2 (testing for the main effect of congruency) and Model 3 (testing for the main effects of congruency and task) were significant [(MM2, (*X^2^* (1) = 11.0, *p* = < .001), (MM3, (*X^2^* (1) = 35.0, *p* = < .001)]. Nevertheless, the comparison indicated that Model 4i with the added interaction of the modality and the task provided the best predictions accuracy of the obtained data considering that it was also the most complex model [(MM4i, (X^2^ (2) = 10.1, *p* = < .01)]. Table 2 below entails the fixed and random effects of Model 4i.

**Table 2:** Linear mixed model fit by maximum likelihood of the fixed and random effects of Model 4i.

**FIXED EFFECTS**							
**EFFECT**	**Mean difference (ms)**	**95% CI**		***df***	***t***		***p***
**(INTERCEPT)**	944	[802, 1085]		20	13.7		< .001***
**INCONGRUENT**	36	[15, 57]		2959	3.4		< .001***
**PLEASANTNESS**	34	[5, 64]		2959	2.3		.021*
**VISUAL**	-48	[–77, –18]		2959	-3.2		.001**
PLEAS.*VISUAL	57	[16, 98]		2959	2.7		.007*
**RANDOM EFFECTS**							
**GROUPS**	**Name**		**Std.Dev.**				
**SUBJECT**	Intercept		296				
**RESIDUAL**			288				
Number of observations: 2978, Subjects: 19							

The estimates of the effect of congruency, task, modality, and the interaction (task*modality) are presented in reference to the intercept in milliseconds. 95% CI = 95% confidence intervals, df = degrees of freedom (Satterthwaite-method), Std.Dev. = standard deviation, t = t-statistics, p = probability.

Significance levels

* p < .05

** p < .01

*** p < .001.

**Fig 2:**
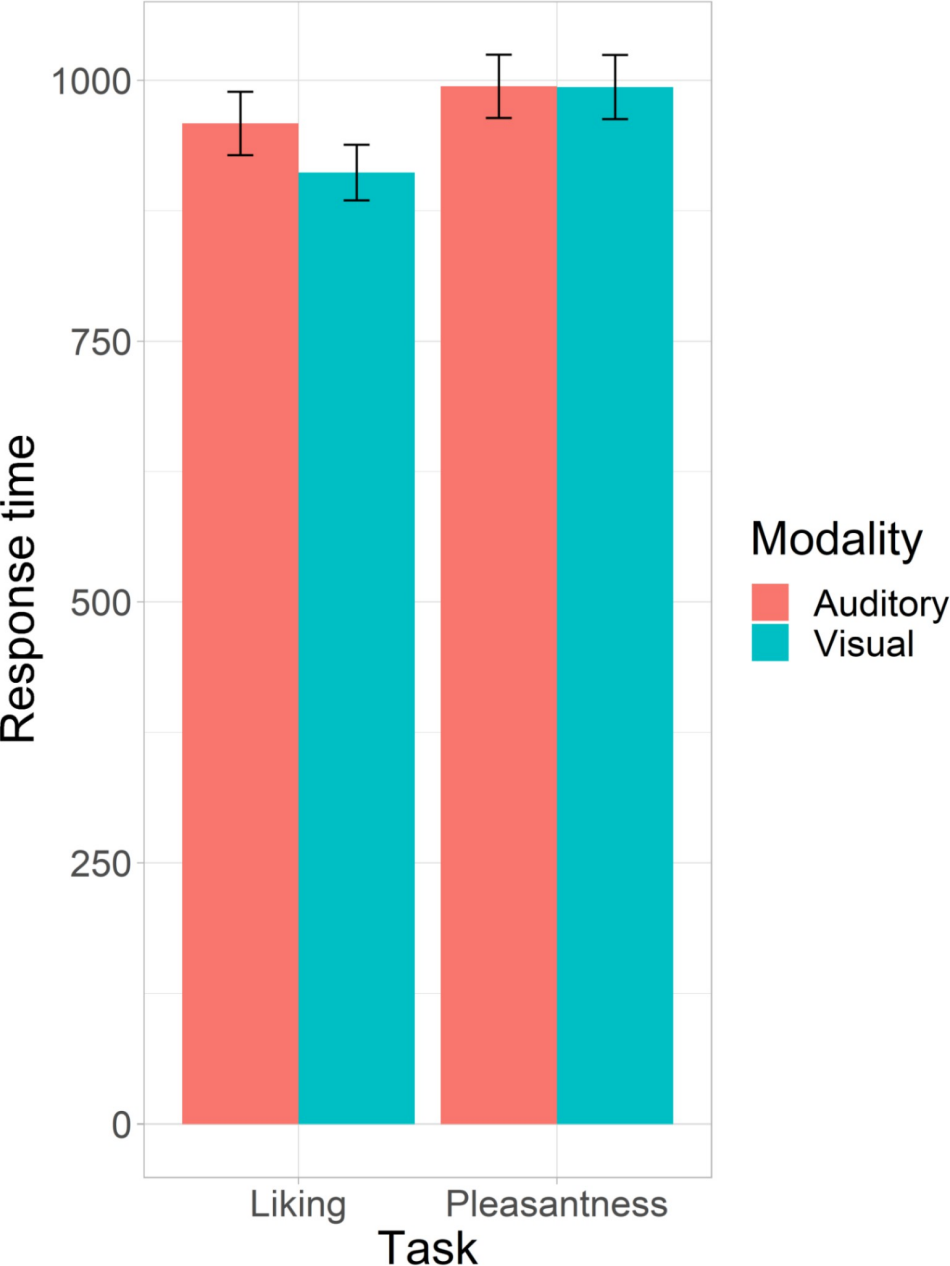
Bar plot of the response times. Average response times (milliseconds) to both tasks in both modalities with error bars of 95% confidence intervals.

**Fig 3:**
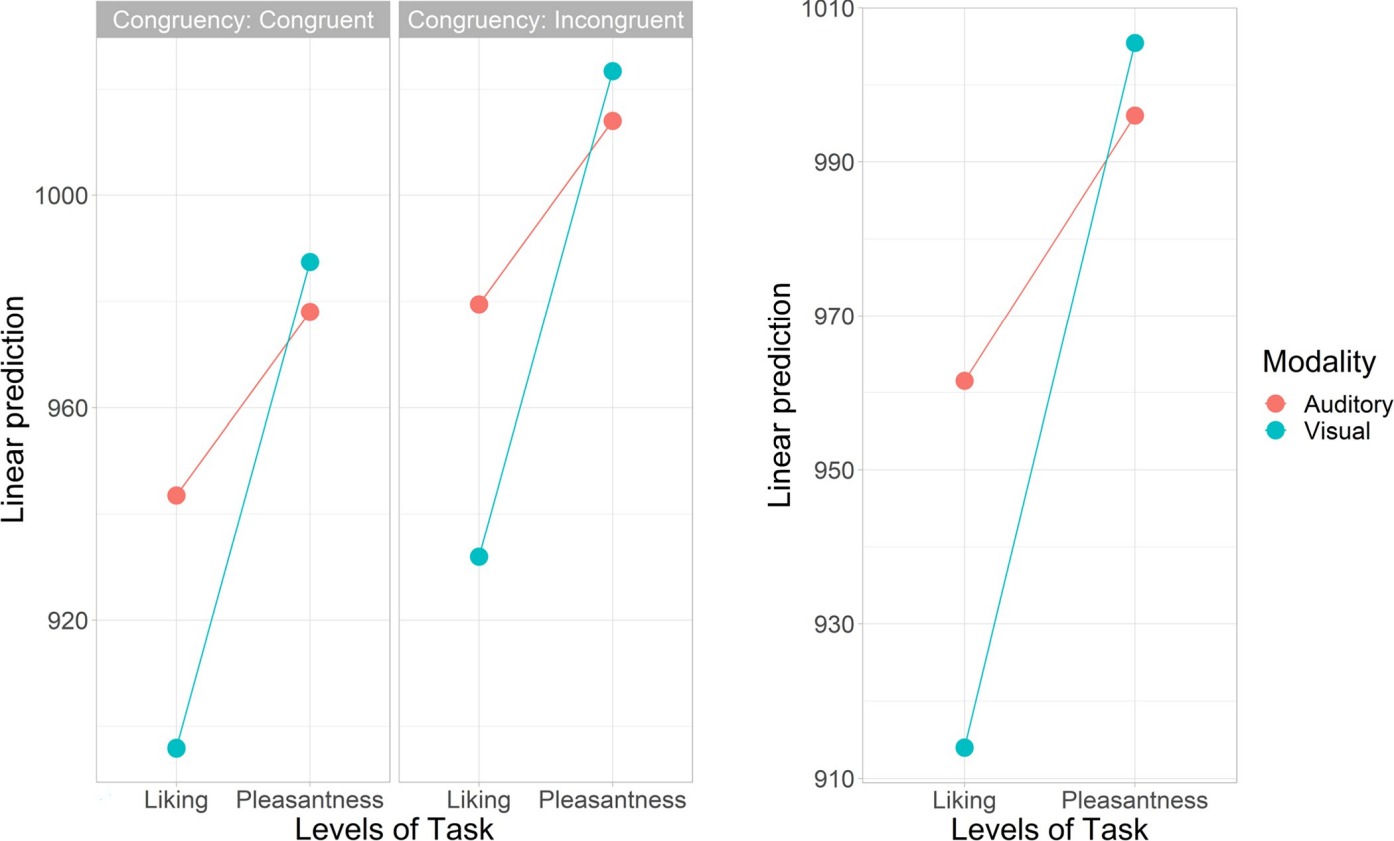
Linear predictions of the response times. Linear predictions for the priming effect in dependence to the sensory modality and task for congruent and incongruent conditions (left), and across congruency (right). The y-axis indicates the response time in milliseconds.

Together with likelihood ratio test and the subsequent Tukey-adjusted pairwise estimation of marginal means computed on the Model 4i (Tables 3 and 4), the results demonstrated that across both modalities, the pleasantness rating was the slowest with no significant difference between the modalities (Fig 2). Instead, the liking rating differed significantly between the modalities being the fastest in the visual modality (for terminological clarification, henceforth the modality is defined by the target. i.e., *visual modality* means that the target is visual). Furthermore, congruency was averaged across the levels of task, showing a shorter latency for the congruent conditions, thus indicating a clear cross-modal affective priming effect (Incongruent-Congruent: *M_diff_* = 36 ms, 95% CI: [15, 57], *df* = 2959, *t* = 3.4, *p* = < .001) (Fig 3).

**Table 3:** Pairwise differences of modalities within tasks and pairwise differences of tasks within modalities of model 4i.

	Contrast	*M_diff_ (ms)*	SE	*df*	*T*	*p*
**PLEASANTNESS**	Auditory—Visual	- 9.4	15.0	2963	- 0.63	.529
**LIKING**	Auditory—Visual	47.5	14.9	2963	3.20	.001**
**VISUAL**	Liking—Pleasantness	- 91.5	15.0	2963	- 6.1	< .001**
**AUDITORY**	Liking—Pleasantness	- 34.5	14.9	2963	-2.31	.021*

Results are averaged over the levels of congruency (for task and modality), modality (for task) and task (for modality).

M_diff_ = Mean difference in response time in milliseconds, SE = Standard error, df = Degrees of freedom (Satterthwaite-method), t = t-statistics, p = probability.

**Table 4:** Estimated marginal means for Model 4i.

	Auditory				Visual		
Task	emmeans	95% CI	df	Task	emmeans	95% CI	df
Liking	962	[818, 1105]	19.7	Liking	914	[771, 1057]	19.7
Pleasantness	996	[853, 139]	19.7	Pleasantness	1005	[862, 1149]	19.7

The table entails the estimated marginal means for each task in the corresponding modality.

Emmeans = Estimated marginal means, 95% CI = 95% Confidence Interval, df = degrees of freedom.

## Discussion

In this study, it was investigated whether a behavioural separation of pleasantness from liking takes place already in the early stages of sensorial feature analysis, and whether the response time depends on the stimulus modality. This was addressed by comparing the response times between liking and pleasantness rating tasks, in response to both visual and auditory targets. Also, it was investigated whether a priming effect takes place across different types of affective pieces of information. This was addressed by measuring the response latencies of emotionally congruent and incongruent prime-target pairs. Here, representational visual or auditory prime stimuli were followed by non-representational visual or auditory target stimuli. Thus, emotionally congruent pairs had positive primes and positive targets, while emotionally incongruent pairs had either positive or negative primes, followed by an opposing target valence.

Contrary to the hypothesis, in both modalities the liking rating was faster than the pleasantness rating; yet, the response latency was significantly shorter in the visual liking task than in the auditory liking task. Thus, a significant interaction effect was found between the task and the sensory modality. This means that providing the liking rating to the geometric visual patterns was the easiest of all the conditions, whereas the pleasantness judgement of the same visual stimuli was least straight forward. Despite the unexpected difference in the response times between liking and pleasantness ratings, the expectation of a bi-directional cross-modal affective priming effect in both sensory modalities took place. This means that representational affective auditory information can facilitate the processing of subsequent non-representational visual affective information, and vice versa. This result indicate that the different types of pieces of affective information, representational and non-representational, seem to share a similar psychological emotion processing mechanism, despite one consisting of emotionally evoking real-life sceneries (primes), and the other consisting of abstract and aesthetic patterns and sounds (targets). Indeed, priming effect took place under conditions of the partially simultaneous presentation of the primes and targets, demonstrating that the participants were able to focus on the targets, despite the overlap in the stimulus presentation.

This study provides evidence for a behavioural delineation of the early affective processes by demonstrating that the applied tasks underlie different response times. Nevertheless, our prediction regarding the tasks was not confirmed as it was expected that the pleasantness rating, which is known largely to rely on rapid feature detection, would be faster. Instead, based on the finding of liking response being the fastest, the results seem to support *core affect* understanding of emotion, where pleasure and hedonic valence are considered to underlie a core *liking reaction* to a stimulus [3], as well as the idea of dimensions of disliking and liking being crucial properties of unconscious emotion perception [6]. Indeed, it has been suggested that humans have affective information about their homeostatic state available, either via feedback from one´s body or from objects or events related to the current environment, giving rise to pleasure or displeasure [1]. Thus, we suggest that perhaps the liking judgement derives information from the person´s homeostatic state, making the liking rating faster, as if the access to the internal information would be more rapid than the sensorial feature analysis, which the pleasantness rating is, at least partially, known to rely on (consonance and dissonance in music, and complexity and symmetry in visual aesthetics) [22, 26, 39].

In this study, the affective rating task was targeted at providing evaluations on aesthetic stimuli, and as such, the results can have implications when constructing or refining models and theories of art engagement and enjoyment. Firstly, the novelty of the results lies in the delivered behavioural measures which demonstrate that refined affective processes can be delineated, not only neuronally but also behaviourally, in a time frame of early perceptual processes (1 to 2 seconds upon stimulus perception), as suggested in a chronometry model on aesthetic experiences [19]. In this model of aesthetic experience, a conscious liking response was placed at about 1000 ms of stimulus onset, based on electrophysiological responses. Furthermore, the results provide further evidence for the susceptibility of affective assessments, next to studies postulating that the evaluation of different objects can be modified depending on whether they are framed as art [12, 13]. These results imply that such affective shifts can be manipulated also in the regions of 1 to 2 seconds by changing the affective task. In particular the results indicate that the cognitive-affective schemata, assumed to be involved in evaluation formation, can also be consciously adjusted for relatively simple features, such as the here applied target stimuli, which have been shown to be rather universal in the evoking of negative and positive associations.

In order to place the results presented here in the context of emotion regulation, a follow-up question would be whether the timing difference would persist when providing ratings to more complex stimuli as well. Finally, when considering the applicability of the results, one should bear in mind that here the participants were pre-given tasks (liking and pleasantness), which then indicated different processing times. This renders the follow-up question whether this difference persists in a natural environment, and whether features such as personality, art expertise or emotion regulatory strategies would influence the results.

## Limitations

The response time difference between the modalities is assumed to be explained by different perceptual encoding and physiology of vision and audition. Audition is performed serially, whereas vision can function in parallel, possibly enabling faster processing of the stimulus. To understand the sensory modality-based differences in more detail the experiment could be replicated with a single-modality protocol, such that primers and targets would be of the same sensory modality. In this study the design choice of conducting the experiment multimodally allowed us firstly, to test whether there would be an affective priming effect between the sensory modalities and secondly, whether it takes place with stimuli representing representational and non-representational affective information. Indeed, the results indicate that the affective processing, as defined by the two applied tasks, takes place across the sensory modalities and across the presentative and non-presentative emotional content. Furthermore, the applied prime-target pairs could be swapped, such that the non-representational stimuli are used as primes and vice versa. This could reveal whether the affect attribution, as defined by priming, takes place similarly also from the non-representative to the representative stimuli. As a further follow-up study, to obtain electrophysiological correlates of the multimodal priming effect, the authors have applied a simplified version of the protocol applied here to further investigate the time course and the affective transfer effect between the sensory modalities (study in review).

A further limitation of the chosen experiment design is related to the task variation; it is not possible to control the attentional attendance of the participants, and whether they really focused on the given task. To minimize participant related variability in the response times, the task varied only per each block, not per trial, so that any possible confusion regarding the task could be avoided. Furthermore, each trial ended with the prompt to remind the participant of the requested task. Furthermore, attendance induced deviations in the data were minimized by excluding abnormally short or long response times.

Furthermore, despite the obtained robust effect sizes, the interpretation of the results should be done in reference to the sample size of 20 participants. Ideally, the study could be replicated with a larger sample size.

## Conclusion

Conclusively, these results demonstrate that the formation of an affective judgement can incorporate different types of affective information across two sensory modalities, as indicated by the priming effect. Furthermore, the results demonstrate that the cognitive framing towards the given task, as defined by the verbal instructions of the experimenter, is encoded into the behavioural judgement already at a very early stage. This is consistent with the hypothesis that liking and pleasantness ratings draw on different resources while guiding the formation of the affective rating. Conclusively, it seems that the delineation of affective processes underling liking and pleasantness is not only related to emotion regulation in longer time span of minutes, but already takes place in the regions of milliseconds when encoded into the intention of the given task.

## Supporting information

S1 Fig Visual positive target stimuli. Starting at the number in the upper left column, each number refers to the corresponding black and white pattern below. The patterns are based on Jacobsen & Höfel, 2002.
(TIF)

S2 Fig Visual negative target stimuli. Starting at the number in the upper left column, each number refers to the corresponding black and white pattern below. The patterns are based on Jacobsen & Höfel, 2002.
(TIF)

S1 Table Rating of the visual positive primes. Valence mean 7.17, standard deviation 0.75. Arousal mean 5.43, standard deviation 1.02.
(DOCX)

S2 Table Rating of the visual negative primes. Valence mean 2.51, standard deviation 0.56. Arousal mean 5.40, standard deviation 0.52.
(DOCX)

S3 Table Rating of the auditory positive primes. Valence mean 6.80, standard deviation 0.59. Arousal mean 5.85, standard deviation 0.79.
(DOCX)

S4 Table Rating of the auditory negative primes. Valence mean 2.55, standard deviation 0.57. Arousal mean 6.74, standard deviation 0.73.
(DOCX)

S5 Table Auditory targets. Right columns show the positive chords, and the left column shows the negative chords.
(DOCX)

S6 Table Experimental design–session structure. Starting from the left column: The table contains the trial blocks, number of trials in each block, the function of the block for the experiment, and the given task in reference to the left and right buttons of the answering device.
(DOCX)

S7 Table Model 1. The following applies to all models presented in S7 to S13 Tables: The estimates of the effect of congruency, task, modality, and possible interactions are presented in reference to the intercept in milliseconds. ES = standard error of the estimate, df = degrees of freedom, Pr = probability, df = degrees of freedom (Satterthwaite-method), t = t-statistics, p = probability, Std.Dev. = standard deviation. Significance levels: * p < .05, ** p < .01, *** p < .001.
(DOCX)

S8 Table Model 2. (DOCX)

S9 Table Model 3. (DOCX)

S10 Table Model 3i. (DOCX)

S11 Table Model 4. (DOCX)

S12 Table Model 4i. (DOCX)

S13 Table Model 4ib. (DOCX)
