# Supplementary figures and images for: I know what i like when i see it: Likability is distinct from pleasantness since early stages of multimodal emotion evaluation
Source: PLoS One. 2022 Sep 13;17(9):e0274556. doi: 10.1371/journal.pone.0274556 (PMC9469973; doi:10.1371/journal.pone.0274556)

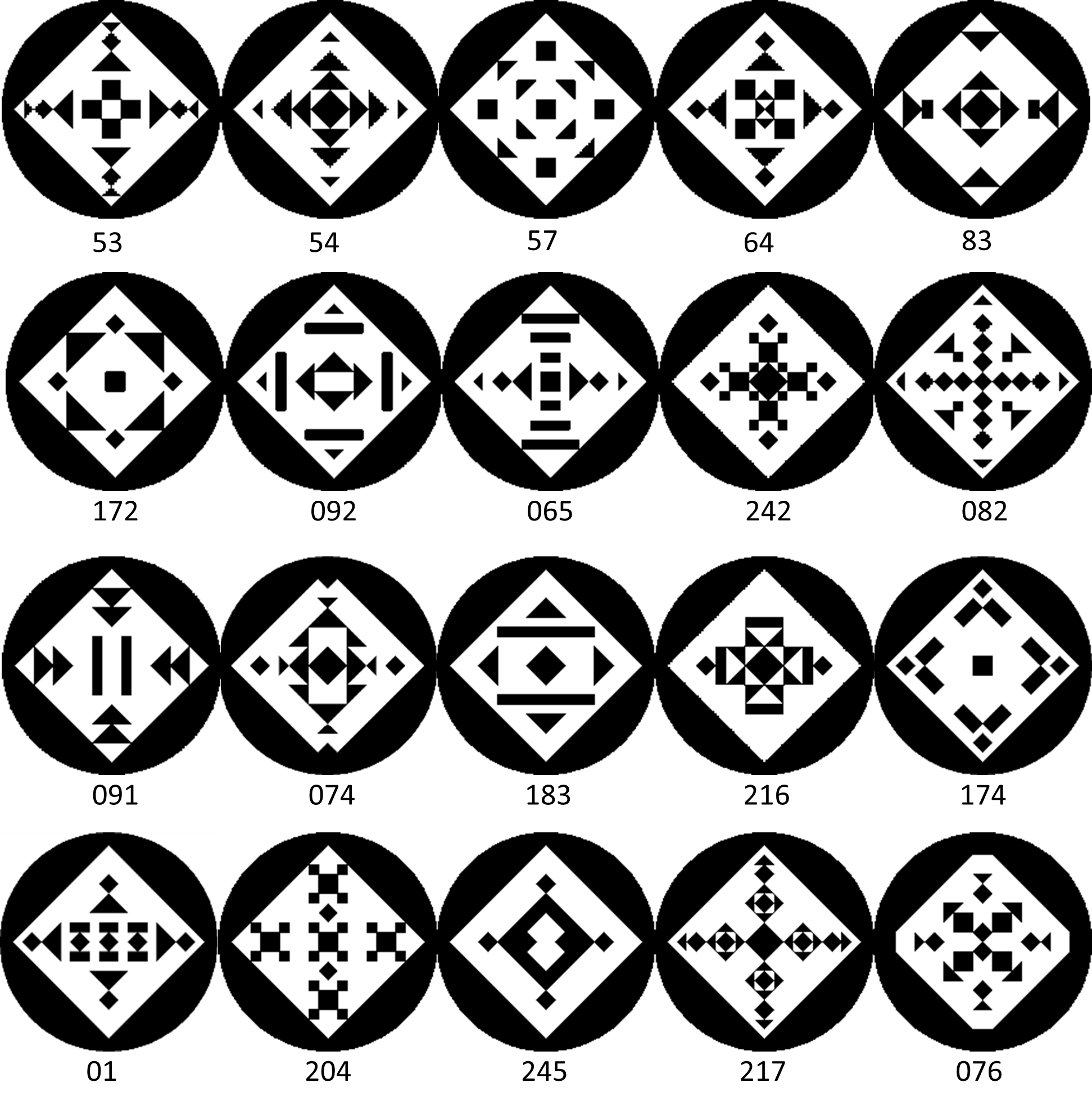

Supplement: S1 Fig — Starting at the number in the upper left column, each number refers to the corresponding black and white pattern below. The patterns are based on Jacobsen & Höfel, 2002. (TIF) [file pone.0274556.s001.tif]

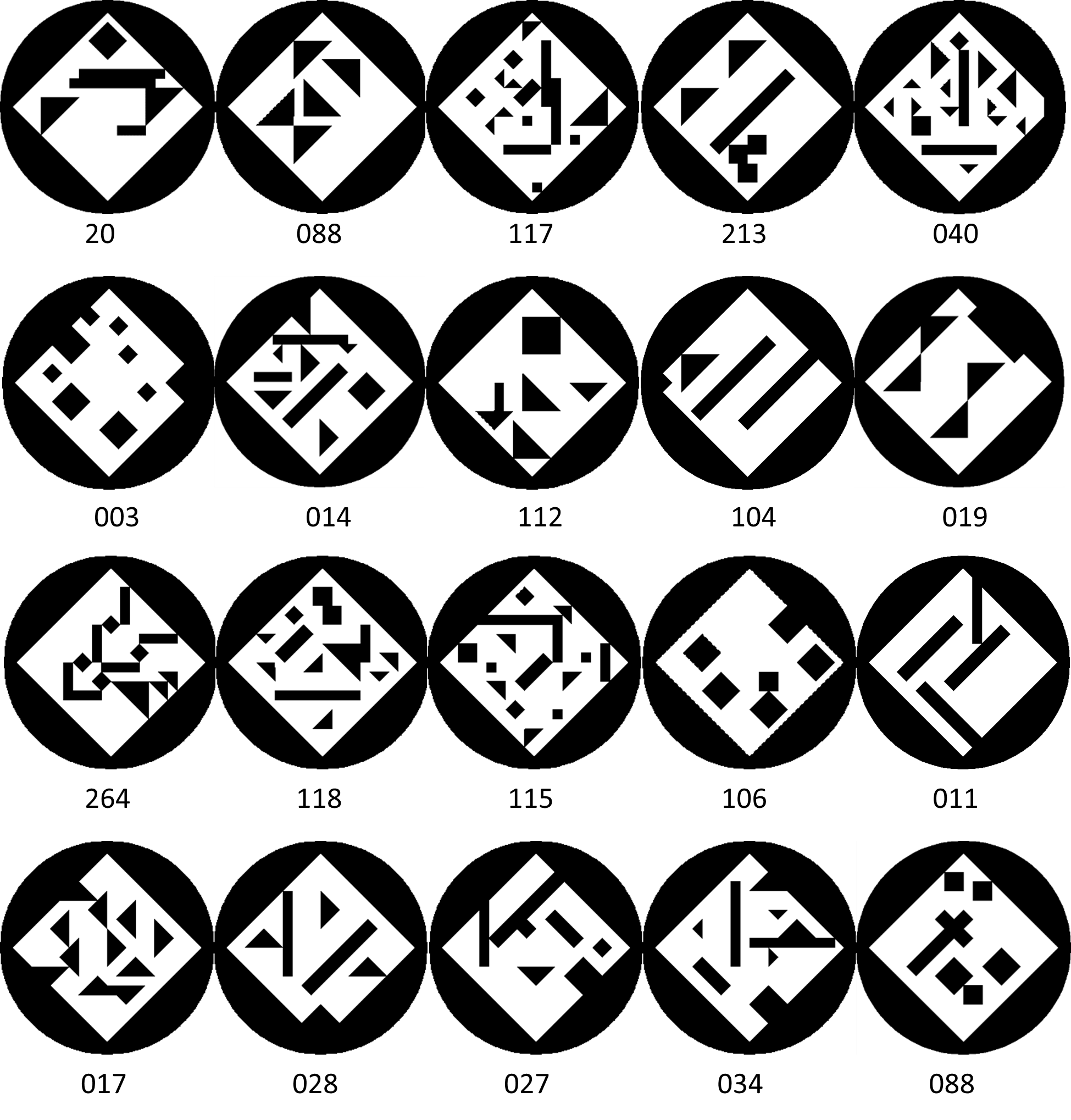

Supplement: S2 Fig — Starting at the number in the upper left column, each number refers to the corresponding black and white pattern below. The patterns are based on Jacobsen & Höfel, 2002. (TIF) [file pone.0274556.s002.tif]
