# Supplementary material for: I know what i like when i see it: Likability is distinct from pleasantness since early stages of multimodal emotion evaluation
Source: PLoS One. 2022 Sep 13;17(9):e0274556. doi: 10.1371/journal.pone.0274556 (PMC9469973; doi:10.1371/journal.pone.0274556)
Supplement: S1 Table — Valence mean 7.17, standard deviation 0.75. Arousal mean 5.43, standard deviation 1.02. (DOCX) [file pone.0274556.s003.docx]

| IAPS nr. | Valence mean (SD) | Arousal mean (SD) | Theme |
| --- | --- | --- | --- |
| 8031 | 6.76 (1.39) | 5.58 (2.24) | Skier |
| 8502 | 7.51 (1.72) | 5.78 (2.49) | Money |
| 1440 | 7.96 (1.59) | 4.76 (2.25) | Seal |
| 2345 | 6.91 (1.59) | 4.60 (2.10) | Children |
| 4626 | 7.36 (1.51) | 5.45 (2.28) | Wedding |
| 4695 | 7.37 (1.31) | 7.00 (1.57) | Erotic couple |
| 5700 | 7.54 (1.56) | 5.44 (2.38) | Mountains |
| 7508 | 7.03 (1.57) | 5.06 (2.21) | Ferris Wheel |
| 1650 | 6.80 (2.14) | 6.40 (1.99) | Jaguar |
| 1710 | 8.02 (1.21) | 5.53 (2.07) | Puppies |
| 1722 | 6.85 (1.55) | 4.65 (2.07) | Jaguar |
| 2655 | 6.62 (1.47) | 4.15 (1.99) | Child |
| 4520 | 4.76 (0.86) | 2.68 (1.77) | Erotic male |
| 4660 | 7.63 (1.30) | 6.92 (1.74) | Erotic couple |
| 5626 | 6.62 (2.34) | 5.98 (2.11) | Hang glider |
| 8300 | 6.54 (1.66) | 6.26 (2.17) | Pilot |
| 8461 | 7.54 (1.35) | 4.54 (2.20) | Happy Teens |
| 8470 | 7.94 (1.31) | 5.98 (2.20) | Gymnast |
| 8496 | 7.94 (1.75) | 6.38 (2.14) | Water slide |
| 8499 | 7.70 (1.36) | 5.56 (2.61) | Rollercoaster |
