# Supplementary material for: I know what i like when i see it: Likability is distinct from pleasantness since early stages of multimodal emotion evaluation
Source: PLoS One. 2022 Sep 13;17(9):e0274556. doi: 10.1371/journal.pone.0274556 (PMC9469973; doi:10.1371/journal.pone.0274556)
Supplement: S2 Table — Valence mean 2.51, standard deviation 0.56. Arousal mean 5.40, standard deviation 0.52. (DOCX) [file pone.0274556.s004.docx]

| IAPS nr. | Valence mean (SD) | Arousal mean (SD) | Theme |
| --- | --- | --- | --- |
| 3225 | 1.82 (1.22) | 5.95 (2.46) | Mutilation |
| 9140 | 2.19 (1.37) | 5.38 (2.19) | Cow |
| 9425 | 2.67 (1.44) | 5.92 (2.13) | Assault |
| 2352.2 | 2.09 (1.50) | 6.25 (2.10) | Bloody kiss |
| 3225 | 2.06 (1.24) | 5.39 (2.41) | Mutilation |
| 5971 | 4.35 (1.64) | 6.43 (2.02) | Tornado |
| 6243 | 2.80 (1.61) | 5.60 (2.34) | Aimed Gun |
| 8485 | 3.23 (1.71) | 6.63 (1.97) | Fire |
| 9140 | 2.56 (1.42) | 4.90 (2.29) | Cow |
| 3160 | 2.63 (1.23) | 5.35 (1.79) | Eye disease |
| 3181 | 2.30 (1.43) | 5.06 (2.11) | Battered fem |
| 6021 | 2.21 (1.51) | 6.06 (2.38) | Assault |
| 6250_1 | 2.83 (1.79) | 6.54 (2.61) | Aimed gun |
| 6560 | 2.16 (1.41) | 6.53 (2.42) | Attack |
| 9007 | 2.49 (1.41) | 5.03 (2.32) | Needles |
| 9300 | 2.26 (1.76) | 6.00 (2.41) | Dirty |
| 9428 | 2.31 (1.31) | 5.66 (2.41) | Assault |
| 9520 | 2.46 (1.61) | 5.41 (2.27) | Kids |
| 9621 | 3.22 (1.76) | 5.76 (2.05) | Ship |
| 9810 | 2.09 (1.78) | 6.62 (2.26) | KKKrally |
| 9910 | 2.06 (1.26) | 6.20 (2.16) | Car accident |
