# Supplementary material for: I know what i like when i see it: Likability is distinct from pleasantness since early stages of multimodal emotion evaluation
Source: PLoS One. 2022 Sep 13;17(9):e0274556. doi: 10.1371/journal.pone.0274556 (PMC9469973; doi:10.1371/journal.pone.0274556)
Supplement: S3 Table — Valence mean 6.80, standard deviation 0.59. Arousal mean 5.85, standard deviation 0.79. (DOCX) [file pone.0274556.s005.docx]

| IADS nr. | Valence mean (SD) | Arousal mean (SD) | Theme |
| --- | --- | --- | --- |
| 109 | 6.40 (2.13) | 5.64 (1.84) | Carousel |
| 202 | 6.81 (2.08) | 7.13 (1.89) | Erotic female |
| 221 | 6.56 (1.75) | 5.05 (1.91) | Male laugh |
| 230 | 7.05 (1.44) | 4.84 (1.86) | Giggling |
| 254 | 6.17 (1.65) | 5.58 (1.99) | Video game |
| 353 | 7.38 (1.53) | 6.62 (1.42) | Baseball |
| 378 | 6.06 (2.01) | 6.15 (2.22) | Doorbell |
| 721 | 6.71 (1.75) | 5.00 (2.12) | Beer |
| 110 | 7.64 (2.10) | 6.03 (1.98) | Baby |
| 111 | 6.01 (2.19) | 5.65 (1.91) | Music Box |
| 151 | 7.29 (1.65) | 4.49 (2.45) | Robin |
| 215 | 5.77 (2.18) | 7.32 (1.57) | Erotic couple |
| 220 | 7.64 (1.86) | 6.01 (2.15) | Boy laugh |
| 224 | 6.74 (1.72) | 5.93 (1.77) | Kids |
| 226 | 7.90 (1.31) | 5.42 (2.26) | Lauging |
| 355 | 6.90 (1.87) | 6.31 (2.10) | Crowd |
| 365 | 6.36 (1.80) | 6.56 (1.62) | Party |
| 716 | 6.83 (2.19) | 6.06 (2.35) | Slot machine |
| 717 | 7.22 (1.76) | 6.57 (1.96) | Slot machine |
| 725 | 6.52 (1.84) | 4.67 (2.05) | Soda fizz |
