# Supplementary material for: I know what i like when i see it: Likability is distinct from pleasantness since early stages of multimodal emotion evaluation
Source: PLoS One. 2022 Sep 13;17(9):e0274556. doi: 10.1371/journal.pone.0274556 (PMC9469973; doi:10.1371/journal.pone.0274556)
Supplement: S4 Table — Valence mean 2.55, standard deviation 0.57. Arousal mean 6.74, standard deviation 0.73. (DOCX) [file pone.0274556.s006.docx]

| IADS nr. | Valence mean (SD) | Arousal mean (SD) | Theme |
| --- | --- | --- | --- |
| 115 | 1.98 (1.32) | 7.20 (1.91) | Bees |
| 241 | 2.27 (1.46) | 6.17 (1.99) | MaleCough |
| 283 | 2.67 (1.60) | 6.19 (1.69) | Fight |
| 284 | 1.64 (0.98) | 7.36 (1.85) | Attack |
| 295 | 3.14 (2.47) | 6.02 (2.00) | Couple sobbing |
| 296 | 2.01 (1.34) | 6.33 (1.90) | Women crying |
| 423 | 2.73 (1.45) | 6.36 (1.75) | Injury |
| 624 | 2.74 (1.81) | 7.09 (1.72) | Air raid |
| 105 | 3.24 (2.32) | 6.27 (2.02) | Puppy |
| 106 | 3.65 (1.74) | 6.27 (1.52) | Growl |
| 242 | 3.02 (1.70) | 5.08 (1.77) | Female cough |
| 255 | 2.49 (1.73) | 5.80 (2.09) | Vomit |
| 261 | 2.61 (2.78) | 6.51 (1.87) | Baby cry |
| 276 | 2.04 (1.38) | 7.65 (1.65) | Female scream |
| 278 | 1.78 (1.57) | 7.16 (2.11) | Child abuse |
| 285 | 1.87 (1.50) | 7.49 (1.91) | Attack |
| 286 | 2.04 (1.46) | 7.69 (1.69) | Victim |
| 424 | 2.74 (1.94) | 7.68 (1.74) | Car wreck |
| 711 | 3.23 (1.67) | 7.45 (1.77) | Siren |
| 719 | 3.09 (1.71) | 6.99 (1.80) | Dentist drill |
