# Supplementary material for: I know what i like when i see it: Likability is distinct from pleasantness since early stages of multimodal emotion evaluation
Source: PLoS One. 2022 Sep 13;17(9):e0274556. doi: 10.1371/journal.pone.0274556 (PMC9469973; doi:10.1371/journal.pone.0274556)
Supplement: S5 Table — Right columns show the positive chords, and the left column shows the negative chords. (DOCX) [file pone.0274556.s007.docx]

| Auditory positive targets | Auditory negative targets |
| --- | --- |
| low_major_3_As_D_F | low_minor_3_D_Fis_B |
| low_major_3_B_Ds_Fs | low_minor_3_E_Gis_Cis |
| low_major_3_C_E_G | low_minor_3_F_A_D |
| low_major_3_Cs_F_Gs | low_minor_3_Fis_Ais_Dis |
| low_major_3_D_Fs_A | low_minor_3_Dis_Gis_B |
| low_major_3_E_Gs_B | low_minor_3_G_C_Dis |
| low_major_3_F_A_C | low_minor_3_Gis_Cis_E |
| low_major_3_G_B_D | low_minor_3_Ais_D_G |
| low_major_3_A_Cis_E | low_minor_3_Cis_F_Ais |
| low_major_3_Cis_E_A | low_neappenta_5_G_Gis_B_Cis_E |
| low_major_3_D_F_Ais | low_neappenta_5_Gis_A_C_D_F |
| low_major_3_Dis_Fis_B | low_neappenta_5_A_Ais_Cis_Dis_Fis |
| low_major_3_E_G_C | low_neappenta_5_Ais_B_D_E_G |
| low_major_3_Fis_A_D | low_neappenta_5_B_C_Dis_F_Gis |
| low_major_3_Gis_B_E | low_neappenta_5_C_Cis_E_Fis_A |
| low_major_3_A_C_F | low_neappenta_5_Cis_D_F_G_Ais |
| low_major_3_D_G_B | low_neappenta_5_D_Dis_Fis_Gis_B |
| low_major_3_F_Ais_D | low_neappenta_5_Dis_E_G_A_C |
| low_major_3_Fis_B_Ds | low_neappenta_5_E_F_Gis_Ais_Cis |
| low_major_3_G_C_E | low_neappenta_5_F_Fis_A_B_D |
