# Supplementary material for: I know what i like when i see it: Likability is distinct from pleasantness since early stages of multimodal emotion evaluation
Source: PLoS One. 2022 Sep 13;17(9):e0274556. doi: 10.1371/journal.pone.0274556 (PMC9469973; doi:10.1371/journal.pone.0274556)
Supplement: S6 Table — Starting from the left column: The table contains the trial blocks, number of trials in each block, the function of the block for the experiment, and the given task in reference to the left and right buttons of the answering device. (DOCX) [file pone.0274556.s008.docx]

| **Block** | **No. of trials** | **Function** | **Items assigned to left-key response** | **Items assigned to right-key response** |
| --- | --- | --- | --- | --- |
| 1 | 10 | Practice | Liking | Disliking |
| 2 | 10 | Practice | Pleasantness | Unpleasantness |
| 3 | 40 | Liking task with visual prime and acoustic target | Liking | Disliking |
| 4 | 40 | Liking task with acoustic prime and visual target | Liking | Disliking |
| 5 | 40 | Pleasantness task with visual prime and acoustic target | Pleasantness | Unpleasantness |
| 6 | 40 | Pleasantness task with acoustic prime and visual target | Pleasantness | Unpleasantness |
