# Supplementary material for: I know what i like when i see it: Likability is distinct from pleasantness since early stages of multimodal emotion evaluation
Source: PLoS One. 2022 Sep 13;17(9):e0274556. doi: 10.1371/journal.pone.0274556 (PMC9469973; doi:10.1371/journal.pone.0274556)
Supplement: S7 Table — The following applies to all models presented in S7 to S13 Tables: The estimates of the effect of congruency, task, modality, and possible interactions are presented in reference to the intercept in milliseconds. ES = standard error of the estimate, df = degrees of freedom, Pr = probability, df = degrees of freedom (Satterthwaite-method), t = t-statistics, p = probability, Std.Dev. = standard deviation. Significance levels: * p < .05, ** p < .01, *** p < .001. (DOCX) [file pone.0274556.s009.docx]

| **Fixed effects** | | | | | | | | |
| --- | --- | --- | --- | --- | --- | --- | --- | --- |
| **Effect** | **Mean difference (ms)** | **ES** | | ***df*** | ***t*** | | ***p*** | |
| **(Intercept)** | 969 | 68 | | 19 | 13.9 | | <.001*** | |
| **Random effects** | | | | | | | | |
| **Groups** | **Name** | | **Std.Dev.** | | |  | |  |
| **Subject** | Intercept | | 295 | | |  | |  |
| **Residual** |  | | 291 | | |  | |  |
| Number of observations: 2978, Subjects: 19 | | | | | | | | |
