# Supplementary material for: I know what i like when i see it: Likability is distinct from pleasantness since early stages of multimodal emotion evaluation
Source: PLoS One. 2022 Sep 13;17(9):e0274556. doi: 10.1371/journal.pone.0274556 (PMC9469973; doi:10.1371/journal.pone.0274556)
Supplement: S10 Table — (DOCX) [file pone.0274556.s012.docx]

| **Fixed effects** | | | | | | | | |
| --- | --- | --- | --- | --- | --- | --- | --- | --- |
| **Effect** | **Mean difference (ms)** | **ES** | | ***df*** | ***t*** | | ***p*** | |
| **(Intercept)** | 911 | 69 | | 20 | 13.3 | | <.001*** | |
| **Incongruent** | 53 | 15 | | 2959 | 3.6 | | <.001*** | |
| **Pleasantness** | 80 | 15 | | 2959 | 5.4 | | <.001*** | |
| **Incong.*Pleas.** | -34 | 21 | | 2959 | -1.6 | | .1 | |
| **Random effects** | | | | | | | | |
| **Groups** | **Name** | | **Std.Dev.** | | |  | |  |
| **Subject** | Intercept | | 295 | | |  | |  |
| **Residual** |  | | 289 | | |  | |  |
| Number of observations: 2978, Subjects: 19 | | | | | | | | |
