# Supplementary material for: I know what i like when i see it: Likability is distinct from pleasantness since early stages of multimodal emotion evaluation
Source: PLoS One. 2022 Sep 13;17(9):e0274556. doi: 10.1371/journal.pone.0274556 (PMC9469973; doi:10.1371/journal.pone.0274556)
Supplement: S11 Table — (DOCX) [file pone.0274556.s013.docx]

| **Fixed effects** | | | | | | | | |
| --- | --- | --- | --- | --- | --- | --- | --- | --- |
| **Effect** | **Mean difference (ms)** | **SE** | | ***df*** | ***t*** | | ***p*** | |
| **(Intercept)** | 929 | 69 | | 20 | 13.5 | | <.001*** | |
| **Incongruent** | 36 | 11 | | 2959 | 3.4 | | <.001*** | |
| **Pleasantness** | 63 | 11 | | 2959 | 5.9 | | <.001*** | |
| **VISUAL** | -19 | 11 | | 2959 | -1.8 | | .07 | |
| **Random effects** | | | | | | | | |
| **Groups** | **Name** | | **Std.Dev.** | | |  | |  |
| **Subject** | Intercept | | 295 | | |  | |  |
| **Residual** |  | | 289 | | |  | |  |
| Number of observations: 2978, Subjects: 19 | | | | | | | | |
