# Supplementary material for: I know what i like when i see it: Likability is distinct from pleasantness since early stages of multimodal emotion evaluation
Source: PLoS One. 2022 Sep 13;17(9):e0274556. doi: 10.1371/journal.pone.0274556 (PMC9469973; doi:10.1371/journal.pone.0274556)
Supplement: S13 Table — (DOCX) [file pone.0274556.s015.docx]

| **Fixed effects** | | | | | | | | |
| --- | --- | --- | --- | --- | --- | --- | --- | --- |
| **Effect** | **Mean difference (ms)** | **ES** | | ***df*** | ***t*** | | ***p*** | |
| **(Intercept)** | 935.5 | 69.5 | | 20 | 13.5 | | <.001*** | |
| **Incongruent** | 52 | 21.1 | | 2959 | 2.5 | | 0.014** | |
| **Pleasantness** | 55.6 | 21.1 | | 2959 | 2.6 | | .008** | |
| **VISUAL** | -48.8 | 21.1 | | 2959 | -2.3 | | .021* | |
| **Incong.*Pleas** | -42.2 | 29.8 | | 2959 | 1.4 | | 0.157 | |
| **Incong.*Vis** | 2.4 | 29.8 | | 2959 | 0.1 | | 0.936 | |
| **Pleas*VIs** | 49.2 | 29.8 | | 2959 | 1.6 | | 0.099** | |
| **INCONG.*Pleas.*VIs.** | 15.5 | 42.2 | | 2959 | 0.4 | | 0.713 | |
| **Random effects** | | | | | | | | |
| **Groups** | **Name** | | **Std.Dev.** | | |  | |  |
| **Subject** | Intercept | | 296 | | |  | |  |
| **Residual** |  | | 288 | | |  | |  |
| Number of observations: 2978, Subjects: 19 | | | | | | | | |
